# Enduring life in between a sense of renewal and loss of courage: lifeworld perspectives one year after hip fracture

**DOI:** 10.1080/17482631.2021.1934996

**Published:** 2021-06-07

**Authors:** Birgit Rasmussen, Claus Vinther Nielsen, Lisbeth Uhrenfeldt

**Affiliations:** a Department of Physio- and Occupational Therapy, Horsens Regional Hospital, Horsens, Denmark; b Department of Public Health, Aarhus University, Aarhus, Denmark; c DEFACTUM, Central Denmark Region, Denmark and Regional Hospital West Jutland, Herning, Denmark; d Faculty of Nursing and Health Sciences, Nord University, Bodø, Norway

**Keywords:** Hip fracture, older people, well-being, physical activity, everyday-life, qualitative, phenomenological-hermeneutic, healthcare provider

## Abstract

**Purpose**: To explore everyday life experiences of being active in aged adults´ with walking impairment one year after hip fracture (HF).

**Methods**: A phenomenological-hermeneutic study design is based on Heidegger´s and Gadamer´s thinking focusing on aged adults being-in-the-world one year after HF. Individual semi-structured interviews were conducted from May to July 2017 in the homes of nine participants, who were part of a longitudinal qualitative study with four interview-rounds for a period of 18 months after the HF event. The analysis was interpretative and secured that the authors´ pre-understanding was put at stake through a five-step process of meaning condensation.

**Results**: One theme, “Enduring life in between a sense of renewal and loss of courage”, described aged adults´ experiences of being active in three sub-themes: “Facing loss and losing courage”, “Taking up the challenge and maintaining courage “, and “Renewing the energy to be active”. Living with being under change and increased vulnerability created a challenge in maintaining courage and reaching for possibilities to unfold their own being in life.

**Conclusion**: This study contributes knowledge on how the aged adults´ experiences of the balance between courage and loss in being active one year after HF are profoundly connected with experiences of well-being.

## Introduction

Being active is vital and for aged adults, it is also a means of preventing falls (Sherrington et al., 2008), maintaining independency, sustaining physical and mental capacities, and being in control (Tak et al., 2013). However, one year after a hip fracture (HF) event, aged adults are known to be inactive (Zusman et al., 2018), which may decrease their well-being and physical and mental health (Vogel et al., 2009) and increase the risk of chronic diseases such as diabetes and cardiovascular disease (Lee et al., 2011). It is acknowledged that motivation for being or becoming more active in one’s everyday life goes beyond the wish to achieve physical and psychological benefits related to physical activity (Puterbaugh, 2009). During the first six months when recovering after HF, striving for well-being through physical activity is found to be a driving force (Rasmussen et al., 2018), including the experience of once again feeling safe and free to participate in everyday activities that matter (Gadamer, 1996). For aged adults who have limitations due to the HF even beyond one year, the experience of being connected to other people supports them in being active and is essential to feeling part of life (Rasmussen et al., 2020). Social relationships are a meaningful and health-promoting context for being active in old age (Holt-Lunstad et al., 2010). However, after HF, aged adults may lack confidence in their own walking ability (Dennett et al., 2012) and become confined to their homes (Bertram et al., 2011). With lost possibilities for socializing, their suffering may increase (Rasmussen & Uhrenfeldt, 2016).

Perceived barriers to being active in aged adults are numerous and include physical pain or discomfort, fear of falling, lack of self-confidence and environmental factors such as bad weather or access difficulties (Baert et al., 2011). A decline in mobility, the ability to manage in daily life, as well as feeling depressed are barriers (Baert et al., 2011) found to be three times higher with HF compared to aged adults without (Bentler et al., 2009). When their functional abilities do not recover after HF, some aged adults struggle with experiences of disappointment (Sims-Gould et al., 2017) or vulnerability (Bruun-Olsen et al., 2018) and with restrictions of their mobility they may experience that being active is connected with hopelessness, joylessness, and a loss of identity (Ziden et al., 2010).

Hip fracture is a serious fall-related event that in 2010, affected around 620.000 people in the European Union and 210.000 people in the USA (Kanis et al., 2012). In Denmark, nearly 7000 aged adults suffer from HF each year. Around 50% of survivors from HF never recover completely (Cauley, 2013; Cauley et al., 2014; Cummings & Melton, 2002; Roche et al., 2005). Although the literature has shown inconsistencies regarding optimal type and duration of rehabilitation (Handoll et al., 2011), evidence is emerging showing that prolonged rehabilitation in the community supports aged adults in recovering (Auais et al., 2012), and psychosocial elements are considered vital to support aged adults in recovering well (Taylor et al., 2010). Still, one year after HF, progress has most likely come to a halt (Magaziner et al., 2015) leaving aged adults to live with permanent loss of functioning, dependence (Dyer et al., 2016; Magaziner et al., 2015), and a fear of falling (Visschedijk et al., 2013). A recent observational study found that around 70% of aged adults never regain their mobility or prior ability to manage daily life activities, such as getting in and out a shower or on and off a toilet, and around 60% permanently lose their ability to independently climb stairs (Tang et al., 2017). The experience of diminished physical, mental and social well-being is mostly prevalent in aged adults who prior to the HF have limitations in their functioning already (Peeters et al., 2016; Tang et al., 2017). When fatigue, pain, decreased mobility, a fear or falling and/or difficulties in managing activities of daily life prevail after HF, aged adults have existential concerns about the impact that the HF has on their whole life (Rasmussen et al., 2018; Rasmussen & Uhrenfeldt, 2016). Lack of progress puts hopes under pressure and they may struggle to find meaning in being active; however, after ending rehabilitation, aged adults tend not to ask for help or additional training (Rasmussen et al., 2018). Experiences of well-being seem to be essential for aged adults to find meaning in being active after HF and are important to consider in healthcare when aiming to enhance functional ability and independency after HF (Rasmussen & Uhrenfeldt, 2016).

### Theoretical framework

In this study, the starting point for understanding the human experience of being active one year after HF is the lifeworld, elaborated by Heidegger as being-in-the-world: the human pre-conscious, connectedness with the world (Heidegger, 1962). “Being-in” means dwelling-in and belonging to the world as a familiar place and according to Heidegger, an experience of creating a space into which one can withdraw within the prevailing challenges of ´the fourfold´ (Heidegger, 1971). As such, the aged adults of this study have challenging experiences of being active within existential conditions such as the ageing body (the sky), the uncertainty of future changes (the earth), a longing for extraordinary encounters with the world, things, and people (the divinities), and the nearness of death (the mortals) (Heidegger, 1971). Being “thrown” into the world (Heidegger, 1962, p. 231), a sense of feeling at home and a sense of permanence and stability are possible even in times where the familiarity of being active in everyday life is challenged by the limitations from a previous HF. The study is based on these ideas and a philosophically founded framework building on Heidegger's writings with different kinds of well-being and suffering unfolded in a dwelling-mobility lattice (K. T. Galvin & Todres, 2011). Aged adults experiences of being active after HF in this study are considered to hold possibilities of dwelling and mobility or a combination of both as part of the interwoven existential dimensions of time, space, body, other people, identity and mood. Dwelling is a capacity to ´be´ settle in the present situation, whereas mobility is a sense of possibility of moving forward towards a new horizon (Todres & Galvin, 2010). Bringing spatiality forward, well-being is possible as a sense of excitement, e.g., for aged adults with HF, the possibility to explore new places on excursions with a day-care centre (mobility) or when a sense of safety exists despite being confined to the home after HF (dwelling) (Rasmussen et al., 2018). Well-being does not eradicate suffering but is a resource and a transcendental possibility available in human life through difficult times. Suffering can be an experience of homelessness, which paradoxically holds the possibility to initiate a striving for feeling at home in one´s situation.

Only few studies on the experiences of aged adults beyond six months after HF exist. Little is known about how the existential challenges in everyday life influence their actions. Specifically, for aged adults who prior to the HF have limitation of their mobility possibilities, well-being and suffering may be at stake. To support aged adults in being active in ways that are needed and wanted, we need knowledge on their challenges and well-being possibilities one year after HF

## Aim

The aim of this study was to explore everyday life experiences of being active in aged adults with walking impairment one year after HF.

## Methods

The study had a qualitative design with individual in-depth interviews anchored in the phenomenological-hermeneutic tradition and included patients from two Danish non-university hospitals (Viborg and Horsens). The design was influenced by Gadamer's reflections on language as a medium of correspondence with experiences of people while researching their factual everyday life (Gadamer, 2013). The study used individual interviews and was the second part of a qualitative longitudinal study aiming to capture experiences of being active at different time-points after HF. The first study used interviews at two weeks and six months after HF to explore barriers and facilitators for being active (Rasmussen et al., 2018). This study investigating experiences one year after HF was directed at experiences of being active at a time-point where loss of mobility and independence were most likely permanent.

### Participants

Participants were included purposefully one year earlier at the hospital where they were admitted with HF. Inclusion criteria defined participants to be aged adults who most likely had experiences of their lifeworld being permanently changed by the HF (Coyne, 1997), that is, 65 years of age or older and living in the community with pre-fracture dependency on walking aids or help (Table I). Of the initial 13 included participants, three had died and one participant dropped out due to a vulnerable mental state. The first author (BR) called the remaining nine participants on the phone approximately six months after the previous interview. All agreed to meet her in their homes again.

**Table I.** Inclusion and exclusion criteria

Inclusion	Hip fracture surgery 65 years or older Dependency on help or on walking aids prior to HF Community living Able to talk about the HF experience
**Exclusion**	Other fracture than HF Discharged to permanent nursing home living

### Researchers

The first author was a licensed physiotherapist and PhD student experienced within in-hospital rehabilitation of aged adults with HF, including cross-sectoral cooperation regarding discharge. The third author, a professor in Clinical Nursing, had research expertise in the fields of application of knowledge and the patient-nurse relation from a patient-oriented perspective. The main focus of the second author, a professor and expert in Clinical Social Medicine and Rehabilitation, was the rehabilitation trajectory. The study was founded by a steering group with the overall purpose of exploring and improving rehabilitation initiatives after HF. The members were leaders from a hospital and from rehabilitation sections in four communities.

### Data collection

Using open, semi-structured individual interviews, data were collected by BR from May to July 2017. The topics were related to the participants´ experiences of changes in being active, their worries and their well-being experiences in everyday life. To further a symmetrical dialogue, BR did not bring an interview guide; however, to maintain focus and create a relaxed atmosphere, initial wordings had been prepared beforehand (Fog, 1998), i.e., “Please tell me about an experience of being active since we last met, that was especially important for you?” “Please, tell me about a good experience, where you were active”, and “Did you have any experiences of being active that caused worries?” The dialogue was building on my previous understanding from having met with participants twice and included questions referring to individual and specific topics from previous interviews, i.e., concerning persons’ worries of being exhausted, or thoughts about limitations in social life. Noticing how they moved about; where and how they sat; things they showed; and places they spoke about in their close vicinity added to a deeper understanding of their possibilities for dwelling and activity and contributed to their verbal descriptions. To establish a relaxed atmosphere, a shift between easy to answer questions that emphasized actions (e.g., “what did you do next?”) and more difficult, value-oriented questions (e.g., “what did it mean to you?”) was applied (Price, 2002). Notes were taken immediately after the interviews. Interviews were recorded on an Apple® smartphone using the Apple app “Memos” and lasted between 35 and 130 minutes.

### Ethical considerations

The study was registered in the Central Denmark Research Council journal nr 1-16-02-422-15 and met the Declaration of Helsinki principles (World Medical Association Declaration of Helsinki, 2013). Participants received no payment for being interviewed. Written informed consent was obtained one year earlier. At the start of the interview, BR emphasized that withdrawal was possible at any time. The interviewer was aware that being familiar with participants from two former interviews, they may reveal more than they intended, a phenomenon Fog (1998) named “The Trojan Horse” (Fog, 1998). Therefore, participants were informed about the option of not answering a question at any time.

### Data analysis

The analysis was an interpretative phenomenological-hermeneutic circular process of transforming participants´ expressions of meaning into a new understanding (Gadamer, 2013). Using a word document to organize data, this study applied an analytical five-step process of meaning condensation (Brinkman & Kvale, 2015). A horizontal and vertical movement through data on each step secured that the authors´ pre-understanding was put at stake in the ongoing movement between the parts and the whole of each interview and of all interviews. When in doubt about how participants´ experiences had changed during the first year after HF, former interviews were re-read (Gadamer, 2013). First, all interviews were transcribed verbatim using the first letter(s) for names and places, noting exclamations, accents, and the length of silences, and indicating emotional expressions such as a trembling voice and laughter. Second, natural meaning units from each interview were condensed into briefer statements and, third, rewritten into a more abstract description of the underlying meaning. In the fourth and fifth step, the existential underpinning of the study was kept in mind. The fourth step interpreted data in relation to the research question. In the fifth step, findings were written into non-redundant themes and sub-themes (Brinkman & Kvale, 2015). Selected excerpts from data illustrated the connection between themes and lived experiences.

### Rigour

Rigour was assured by provoking the researchers´ pre-understanding through the hermeneutical circle. This occurred when using a five-step process of meaning condensation; discussing the partial understanding arising at different steps of the interpretation between the co-authors (Gadamer, 2013); and when preliminary findings were presented in teaching for healthcare providers (HCP) in the communities, using the resonance or lack of resonance with the findings as a hallmark regarding whether existential trustworthiness was achieved (Smythe et al., 2008).

## Findings

Pseudonyms are used throughout when referring to the study participants. The nine participants (Table II) had a mean age of 81 years ranging from 71 to 93. Apart from Frank, who had recently moved into an apartment in nursing home facilities, all were still living in their own homes. For all participants, family was a central part of their lives. Six had close contact with neighbours and all talked about a spouse, children, friends or neighbours helping out with daily life chores. Observations and verbal descriptions revealed that since the previous interview, their functioning had not improved. In four participants, other diseases meant that their mobility had further decreased. Although functioning had not improved, participants had taken up some of their earlier social activities; however, they also abstained from some activities due to new dependency. Six participants joined activities outside the house several times a week, and four were assigned to a social day-care centre, a new event for one participant. Apart from Dorthe, whose walking had improved compared to prior to the HF, all the other participants had more difficulty in walking.

**Table II.** Characteristics of participants

*Pseudonym* *Age/gender*	*Walking aid*	*Residence*	*Marital status and examples of relationships*
Dorthe 75/F	Walker outdoor	Older friendly housing. Town area.	Widow. Children nearby and far away; at least weekly contact; celebrates birthdays and Christmas; help out shopping for big items. Almost daily contact with neighbours. Two weekly days at social day-care centre.
Bodil 79/F	Walker	House. Countryside.	Widow. Living with son and daughter-in-law. Two weekly days at social day-care centre.
Karen 81/F	Walker	Ground level flat.	Widow. Children nearby; shopping together once a week; weekly visit from daughter helping out with small jobs. Daily contact with friend next door. Participates in activities at nursing home.
Margrethe 80/F	Walker outdoor	Apartment, 6 steps up. Town area.	Widow. Children far away; visits, celebrates birthdays and Christmas. Close, male friend visiting three times a week and daily contact on phone.
Joan 86/F	Walker away from the house	Villa. Town area.	Widow. Children nearby; help out gardening; contact several times a week; Befriends other relatives. Telephone contact with neighbour several times a week. Visits from red-cross friend.
Lene 91/F	Walker	Older friendly housing.	Widow. Children far away. A daughter helps out in the house. Participates in numerous activities for elderly. Talks with neighbours and knows a lot of people.
Else 93/F	Walker	Villa. Town area.	Widow. Children far away; visits, celebrates birthdays and Christmas. Regular contact on phone with other family members who visit overnight twice a year. Neighbours are good friends; do the shopping and collect medicine.
Frank 71/M	Wheel-chair	Nursing home since three months ago.	Married. Lives apart from wife; overnight visits at home every weekend. Spend all weekdays in social day-care centre.
Gunnar 78/M	Walker	Villa. Town area, children nearby.	Married. Almost always together with wife; children nearby visiting regularly. Good friends living far away visiting 3–4 times a year. Two weekly days at social day-care centre.

One main theme: “Enduring life in between a sense of renewal and loss of courage” was developed to describe aged adults’ experiences of being active through three sub-themes: “Facing loss and losing courage”, “Taking up the challenge and maintaining courage “, and “Renewing the energy to be active” (Table III).

**Table III.** Main theme and sub-themes

Main theme	Enduring life in between a sense of renewal and loss of courage		
*Sub themes*	Renewing the energy to be active	*Taking up the challenge and maintaining courage*	*Facing loss and loosing courage*

One year after HF, physical progress had come to a halt and increased dependency on help or assistive devices, which were part of life challenges. Living with being under change, facing worsening of chronic diseases, further reduced functioning, and increased vulnerability, participants were aware of living in the nearness of death. It was a challenge to maintain courage and reach for possibilities to unfold their own being in life. They were stretched between experiences of well-being feeling renewed and experiences of suffering losing courage; between these two extremes, endurance was a centre point of balancing. Facing loss, participants were taking up the challenges of everyday life. Bodil was living in the countryside with her son and daughter-in-law and could feel lonely because they often were away from the house. She said:

“ … I cannot muster any optimism […]I felt that way on Monday; and on Tuesday, I went to the day care centre and that helped! Because there were so many other things I had to listen to and talk about and so on. Then the fog lifts […] and I forget to think about all the negative things (Joan).

Finding well-being was essential to avoid losing courage and to renew the energy to continue being active.

### Facing loss and losing courage

With lack of progress and thoughts of eventually living with more mobility restrictions and becoming unable to do what they wanted, participants could lose courage. Bodil living with a son and daughter-in-law talked about her hope of being able to again help out doing household chores as before the HF was disappointed. She had “*black days where nothing mattered!”*. She felt *“it is boring being me”* and was “*lacking energy”*. When the body could no longer be trusted, nor support carefree mobility, it was risky and a struggle to be active. Joan was walking a short distance without her walker and suddenly afraid of falling would think:

“well, how do you place those feet […] and will that make you lose your balance”.

Courage was difficult to maintain when being active meant that the energy loss was greater than the benefits. Lene, earlier accomplishing tasks without giving it a second thought now was facing an eternal chain of exhausting days. The HF caused a rupture to her life and her dreams:

“I feel that I´ve aged too quickly […], all of a sudden now you can´t drive anymore, boom! […] you don´t have the strength to go and rake in your garden; boom! You can´t make your own food; […] little by little, you can´t do it anymore.”

Being forced to give up on previous valued activities was difficult to endure and could disturb one's sense of identity. When in need of help, a fear of being a burden made it difficult to ask for it. Feeling vulnerable and exposed to the judgement of other people and to avoid losing courage, staying at home from activities or conforming to what was easiest for the people helping out could be the result.

For two participants, dependent on help, loss of courage was reinforced by HCP actions or thoughtlessness. Else was feeling imprisoned when they took away her second walker because the municipality now only allowed loans of one walker. She didn't know how to replace it and was no longer able to go out into the garden. Lene had helpers who did not recognize her needs and wishes. Her experience was that help could increase her suffering and deprive her of, rather than maintain, her well-being. She had been assigned extra help to re-organize her clothes; however, the helpers did not fold it the way she wanted. The next time she took a pair of jeans out of the cupboard, all the jeans came out, and now they were in a pile on the floor. She had tried correcting the helper explaining:

“‘[I said] this is how I fold a shirt’. ‘Yeah well, that´s good I guess’ she said, but she kept doing it her own way! […] many people take offence if you say something such as ‘I would like it so and so’“

Helpers felt that she was criticizing them, as if she was “*suggesting they couldn't do anything right*”. Therefore, Lene preferred to either does things on her own risking exhaustion or giving up and leaving it.

### Taking up the challenge and maintaining courage

It was a challenge to maintain hope, enjoy life, and pursue life projects. However, in the face of loss, maintaining courage was possible when feeling protected in a manageable and coherent everyday-life and being able to keep on managing some everyday-life tasks. A fear of falling was prevalent and it was vital to feel safe. This was possible in the interplay between one’s own actions, environment and receiving help from other people. Participants walked slowly, used walking devices, walked together with someone else, and used their creativity to manage everyday tasks. Following routines and carefully organizing actions, e.g., when taking a shower, which was perceived to be a delicate situation, gave a feeling of being less exposed to falling. For Lily, almost blind and walking unsteadily, everyday life mostly took place in her kitchen; she talked about how she had accepted this, and it made her feel safe

“because I´m used to being here and it´s ok. That´s the way it is”.

Taking risks could be necessary; however, moving with caution was becoming integral to being active.

Working hard to be true to one’s own values was a key part of the identity. Jane spoke about how hard she was struggling to keep her house and despite a fear of falling kept on gardening using the weeding tool to lean on. Her children were scolding her when asked during the interview whether she couldn't stop working so hard she replied:

“No I can´t […]. I can and want to do it myself […]. That´s how it´s always been”.

Persistently aiming to be active and do things independently restored a sense of dignity, a feeling of being alive, sound, and normal. Still, managing ordinary household chores was exhausting and a challenge on the edge of what was possible to endure. Lene explained that

“So if you can´t take care of yourself, you have to do it anyway. There´s no yelling ‘can you help me with this, can you help me with that’“.

Exertion was accepted; however, the courage to be active was more easily maintained when reaching for a balance where efforts were consistent with available resources. It was satisfying to use one’s own creativity to figure out how things could be done more easily. Easy access to things, for example, through modification of the home environment, the existence of assistive devices, or shops nearby saved sparse resources, and made it possible to preserve energy to do other tasks or to socialize. Lene had created a system where clothes were left out in the open, because then she could *“find it without having to overexert myself by having to search high and low through drawers and cupboards to find this and that”.*

When everyday life was too hard, remaining independent could mean depending on help from others. Lene was in need of help to get down groceries from shelves at the supermarket:

“I have a particular technique. I keep on trying to reach the stuff I want; when someone is passing I look straight at them, and keep on looking till they ask if they may help me”.

Dignity in dependency was challenging to maintain, however, easier when acknowledging one’s own endurance and persistency. Receiving help from people who knew them in light of their past and their life story was preferred. Karen, now in need of help to take her walker into the car, explained:

“it´s not nice needing help for practically everything […] getting out of the car I need an arm to hold on to […] I get upset […] when, I have always been able to. I´d rather have my own people with me (kids) […] someone I know. They´re easier to talk to, I think, than strangers”.

Hope for progress after HF was still present. Participants were training at home and had thoughts about how best to care for their own health. Karen valued physiotherapy because to her it meant: *“You won’t shrivel up into nothing. Or turn into a vegetable”*. Training with a physiotherapist, knowledgeable and with interpersonal skills gave new knowledge about the body. This nurtured confidence in one’s own mobility and gave hope that they would have less pain and feel safer in the future.

### Renewing the energy to be active

Reaching out for other people and feeling part of a community were essential for maintaining a feeling of being meaningfully alive and keep on being active. This was apparent from specific meaning units in the data analysis and also from the way participants addressed and spoke about other people, such as the photos they showed, phone calls during the interviews, etc. At times during interviews, participants would lack words, but when talking about being active with family and friends, they relaxed and talked with renewed energy. From the experience of sharing with other people, e.g., at music events or going to a day-care centre, focus turned away from limitations and loss, the troublesome everyday life, and the awareness of the nearness of death. For Lene, getting to know people when walking around her neighbourhood and then later on being able to greet them in the street were energizing:

“It means that I’m still considered a human being and not just a sad case […] I’m not just someone running around not knowing where I’m going but I actually know what I´m doing”.

Other people caring to understand their situation gave optimism and a sense that being active was meaningful. This could be experienced in very diverse situations, e.g., a bus driver helping out with the walker while telling about his/her own fracture experiences, or neighbours popping by, helping out with shopping, or decorating for Christmas. Also, small considerate actions towards other people, e.g., serving homemade cookies for helpers and people coming by, confirmed the relation with the world. Joan´s neighbour had been admiring her white Peonies and afterwards she reflected that he would like a bouquet for his wheelchair-bound wife, a friend for many years:

“So I picked a bouquet, went over and said: ‘You can bring this for (female friend’s name) to enjoy’. […] Now I can see through her window it’s on the table in the living room”.

A sense that something new could happen and there was something to look forward to renewed feelings of still being on the move and part of life vicissitudes. This could be ignited from being invited to birthday parties or weekend stays, or from small changes in everyday life such as the possibility of going shopping, or just looking at buildings in one’s hometown. Even simple, everyday experiences such as being in the sun, sensing the fresh air; enjoying a favourite cup of tea by the terrace door; and reading books were energizing. In these moments of perceptual pleasure and complete emersion in activities, the heavy weight of everyday challenges vanished. For Gunnar, the best experience was playing chess with a friend at the day-care centre. As he stated,

“I’m simply away from the outside world”

For Lene, the effort of cutting down Jerusalem Artichokes on her terrace was a struggle but worth the effort.

“That spot is sunny in the morning and when it starts to get warm I can sit and eat my breakfast. When the Jerusalem artichokes grow back, passers-by can see my head but they can’t tell that I’m sitting in my robe”.

Being able to make their own decisions and going about their daily lives gave a sense of freedom. Asking Joan how she was managing, she spoke about how she had been struggling when meeting her a year ago. Bringing my awareness to how this contrasted with her present situation, she with a smile looked around her living room, clean, flowers in a vase, table set for coffee and said:

“well, just as you can see. I have it the way I want it; no one bosses me around”.

Despite persistent limitations in functioning, finding space for dwelling and again feeling independent and in control was possible.

## Discussion

The aim of this study was to understand aged adults’ experiences of being active one year after HF. Todres’ and Galvin's theory of well-being provided a framework to inductively understand the specific context that aged adults were in one year after HF at a deeper level and how well-being and suffering were part of their existence (Todres & Galvin, 2010).

The results underline how aged adults one year after HF are living with continuous loss of functioning and independency. Feeling vulnerable, they are enduring being active in a dynamic interrelated experience of being stretched between experiences of renewal and of loss of courage. These findings add empirical evidence to the theoretical framework of well-being, clarifying how the suffering aged adults’ experience after HF may be overwhelming and push towards inactivity. However, well-being experiences can counterbalance suffering and one year after HF well-being experiences seem to support a sense of still being part of life. The findings indicate that without dwelling, aged adults may be struggling too hard and without mobility, they may limit their own possibilities for being active. Experiences of dwelling and mobility that seem to be essential for aged adults one year after HF are, for example, having small, joyful experiences of nature; making one’s own decisions; acknowledging how dependency on help and assistive devices can allow for independent activity; and talking about things to look forward to.

One year after the HF, it is still relevant to consider whether training focused on movement strategies and quality is relevant for the individual aged adult. The aged adults of this study had experiences of the body betraying them and could not be relied on as before the fracture. Fear of falling is prevalent after HF (Visschedijk et al., 2013) and can mean lack of social contact with increased loneliness as a result (Jellesmark et al., 2012). With a fear of falling, activities become limited and our findings support earlier findings stating that aged adults one year after HF have a need to be careful, accept help, and plan their activities in detail (Ziden et al., 2010). However, a fear of falling can be modified (Whipple et al., 2018) and interventions to reduce a fear of falling can be relevant still one year after HF (Auais et al., 2012).

When aged adults one year after HF experience that they are still unable to embrace a wish to be active as they were before the HF, they may start losing courage and this may lead to staying at home from valued activities. These findings support earlier studies describing feelings of helplessness and isolation (Jellesmark et al., 2012) and feeling lonely (Gesar et al., 2017) as a consequence. The hopelessness that aged adults experience is also a risk of depression (Bruggemann et al., 2007). Unable to fulfil a wish to be active and feel part of life, they may withdraw from activities and slide into feelings of depression (K. Galvin & Todres, 2012). In accordance with other studies, we emphasize that special attention towards signs of depressed mood and hopelessness is called for (Griffiths et al., 2015; Jellesmark et al., 2012; Ziden et al., 2010). A new finding from this study is that loss of courage and feelings of hopelessness is not only a permanent state but can also be either intensified or relieved. Aged adults one year after HF are aware that death is coming closer. However, it seems that they avoid losing courage, and as Heidegger puts it, they avoid “ … darken dwelling by blindly staring towards the end” (Heidegger, 1971, p. 149). They maintain hope and their longings towards still being part of life are closely related to relationships with other people. This finding supports and expands earlier studies pointing to the centrality of support from other people and how other people are encouraging and help the aged adults remain optimistic after HF (Young & Resnick, 2009; Ziden et al., 2010). Though aged adults can have an underlying fear of becoming a burden, the courage to be active and keep on going after HF seems to be deeply rooted in a feeling of being connected with other people. This applies both in terms of mobility, a desire to connect with and get to know the life story of other people, and in terms of dwelling, a sense of belonging with familiar others and being part of a community (K. T. Galvin & Todres, 2011). The connection between social relationships and mortality is well established (Holt-Lunstad et al., 2010), and we underline that the spark to remain active after HF is deeply connected with social relationships. We emphasize the need, in accordance with aged adults’ individual preferences and needs, to support possibilities for intersubjective experiences, also one year after HF.

This study points to the fact that feelings of hopelessness can be reinforced by HCP not recognizing aged adults’ needs. Applying specialist knowledge and community guidelines, but not entering a dialogue recognizing what matters to the individual may lead to aged adults losing possibilities for well-being and for being active. The more vulnerable a person is, the more important it seems to be treated with dignity by HCP, and it has been highlighted that dignity for aged adults entails feelings of being acknowledged, feeling worthy, and being treated as a unique person (Clancy et al., 2020). One year after the fracture, aged adults have experiences of their own vulnerability in terms of being exposed, rather than experiences of being weak and breakable (Pan et al., 2019). Treating aged adults as people more exposed to loss and, however, still capable of making choices and striving to reach a goal is vital. Without such respectful cooperation with HCP, the consequence can be the individual sliding towards experiences of losing courage and becoming inactive.

The expectation of recovering and returning to life as it was before the fracture is dominant in aged adults (Healee et al., 2011; Ziden et al., 2010, 2008). However, in line with other studies, we highlight that one year after HF, many aged adults have to live with permanent loss of mobility and increased dependency. Unrealistic expectations about recovering completely may increase the risk of disappointment and dissatisfaction (Sims-Gould et al., 2017). While struggling and striving for a return to former functioning can be meaningful during the first six months (Rasmussen & Uhrenfeldt, 2016), we underline how, for this particular group with pre-fracture dependency on walking aids or help, keeping on struggling too hard to return to former mobility and independency one year after HF can bring suffering and how settling with new post-fracture limitations can be essential to maintain courage and keep on being active.

It is known that increased dependency is persistent one year after HF and it can be difficult to accept (Sims-Gould et al., 2017; Ziden et al., 2010). However, for the aged adults of this study, it could be endured when it was possible to maintain a sense of dignity. In our study, the acknowledgement of one’s own endurance in trying hard to manage without asking for help seems to be essential. In the struggle to preserve dignity, however, there is a risk that when everyday life becomes too exhausting, aged adults lose the courage and energy to be active. In line with a study pointing out that resilience is embedded in physical and social contexts (Wiles et al., 2012), we find that the energy to remain active is extrinsically connected with, as mentioned earlier, relationships with others and additionally with the support and help available in the surroundings. Further, rather than considering independence a matter of managing without help and trusting only in one’s own capability, the application of continuous practical and social support in the home environment seems to be an approach that more genuinely supports aged adults’ possibilities for being active in wanted ways than aiming to withdraw personal assistance in everyday life activities (Bødker et al., 2019). For aged adults, lack of interest from HCP and lack of acknowledgement are found to be a threat to dignity (Clancy et al., 2020). We recommend that HCP supports aged adults by encouraging their acknowledgement of their own persistency despite dependency and their creativity when managing everyday tasks and by assisting them in exploring ways to manage everyday life tasks more effortlessly with or without help. At the same time, we suggest considering how to support an everyday life which is more manageable and where aged adults feel secure and use less effort in activities.

### Strength and limitations

The framework of dwelling-mobility served as a valuable horizon when aiming to understand aged adults´ experiences of being active at a deeper existential level. It was used inductively in the thinking process, and contributed to widening the authors´ understanding, and avoiding prejudices from first author’s experiences from working with rehabilitation as a physiotherapist.

The longitudinal design provided the first author with a familiarity with participants, which could have led to a reproduction of the first author’s subjective knowledge. However, pre-understandings were continuously tested by reflecting with co-authors which enhanced the trustworthiness of the study.

The study included a group of aged adults representing the most vulnerable. It was a limitation that some of the participants were of few words and had difficulties in sharing their reflections. At times, the meaning of the participants´ talk would be embedded in knowledge from previous interviews, which is why selected quotes from the transcripts could be short and fragmented. This was a limitation. However, based on two previous interviews the participants´ stories were interpreted within a temporal perspective. This supported a richer understanding of their lives. Within hermeneutics, understanding is based on interpretation and is a process of developing a new understanding. Depending on the horizon of the author and ongoing awareness towards being open to a new and deeper understanding, a richer understanding is possible to achieve. The horizon of the authors was continuously enlarged from several meetings and from reading literature on illness experiences in aged adults (Gadamer, 2013). Furthermore, the trustworthiness was increased by using a checklist with criteria for qualitative research when reporting the study (Tong et al., 2007).

The prolonged engagement with the same participants is considered a strength as it provided consistency and stability throughout the study (Fleming et al., 2003) and enabled a deeper understanding of the participants’ whole situation. The development of trust and confidentiality allowed for conversations about personal issues, which provided thick descriptions of their lifeworld experiences (Shenton, 2004). This may have increased the quality of the interviews (Brinkmann & Kvale, 2014).

The presence of a close relative during one of the interviews was considered a limitation; however, it turned out to be a strength. Talking about other details and reflecting on changes in their life situation, the relative encouraged the participant to talk more, thereby contributing to a richer account. The relative’s account became part of the researchers´ horizon (Gadamer, 2013) and was useful to better understand the participants´ whole situation when analysing data (Norlyk et al., 2016).

The sample of participants represented variation in age and gender, family relationships and residential status, which was considered a strength. All were living in a country in which homecare services to some extent were tax financed. None of the participants lived in major cities; the women in the study were widowed, and the two men were married. Transferability of study results should be considered within these limits.

## Conclusion

This study contributes with new knowledge regarding aged adults’ experiences of being active one year after HF by describing existential dimensions of their everyday lives. To achieve a sense of well-being, aged adults are enduring being vulnerable as a central part of their being while considering their possibilities for being active. To improve aged adults’ well-being in activity one year after HF, continuous awareness from HCP and healthcare systems towards their possibilities for feeling renewed and avoiding losing courage is needed. Supporting aged adults’ sense of identity and freedom and their possibilities for feeling safe and for being in mutual relationships may allow them to find the energy to be more active. Further, attention is suggested towards whether they are enduring too much or settling for too little; whether the limitations they are enduring are modifiable; whether they are able to settle with losses and limitations not modifiable; and whether they are at risk of losing courage. Including these elements into intervention studies will reveal whether they enhance aged adults´ possibilities for feeling renewed and avoid loss of courage.

## References

[cit0001] Auais, M. A., Eilayyan, O., & Mayo, N. E. (2012). Extended exercise rehabilitation after hip fracture improves patients’ physical function: A systematic review and meta-analysis. *Physical Therapy*, 92(11), 1437–12. 10.2522/ptj.20110274 22822235

[cit0002] Baert, V., Gorus, E., Mets, T., Geerts, C., & Bautmans, I. (2011). Motivators and barriers for physical activity in the oldest old: A systematic review. *Ageing Research Reviews*, 10(4), 464–474. 10.1016/j.arr.2011.04.001 21570493

[cit0003] Bentler, S. E., Liu, L., Obrizan, M., Cook, E. A., Wright, K. B., Geweke, J. F., Chrischilles, E. A., Pavlik, C. E., Wallace, R. B., Ohsfeldt, R. L., Jones, M. P., Rosenthal, G. E., & Wolinsky, F. D. (2009). The aftermath of hip fracture: Discharge placement, functional status change, and mortality. *American Journal of Epidemiology*, 170(10), 1290–1299. 10.1093/aje/kwp266 19808632 PMC2781759

[cit0004] Bertram, M., Norman, R., Kemp, L., & Vos, T. (2011). Review of the long-term disability associated with hip fractures. *Journal of the International Society for Child and Adolescent Injury Prevention*, 17(6), 365–370. 10.1136/ip.2010.029579 21486987

[cit0005] Bødker, M. N., Christensen, U., & Langstrup, H. (2019). Home care as reablement or enabling arrangements? An exploration of the precarious dependencies in living with functional decline. *Sociology of Health & Illness*, 41(7), 1358–1372. 10.1111/1467-9566.12946 31020676

[cit0006] Brinkman, S., & Kvale, S. (2015). *Interviews: Learning the craft of qualitative research interviewing* (3rd ed.). Sage Publications.

[cit0007] Brinkmann, S., & Kvale, S. (2014). *Interviews: Learning the craft of qualitative research interviewing* (3rd ed.). Sage Publications.

[cit0008] Bruggemann, L., Nixon, R. D., & Cavenett, T. (2007). Predicting acute anxiety and depression following hip fracture. *Journal of Behavioral Medicine*, 30(2), 97–105. 10.1007/s10865-006-9088-x 17252203

[cit0009] Bruun-Olsen, V., Bergland, A., & Heiberg, K. E. (2018). “I struggle to count my blessings”: Recovery after hip fracture from the patients’ perspective. *BMC Geriatrics*, 18(1), 18. 10.1186/s12877-018-0716-4 29351770 PMC5775577

[cit0010] Cauley, J. A. (2013). Public health impact of osteoporosis. *Journals of Gerontology Series A: Biomedical Sciences and Medical Sciences*, 68(10), 1243–1251. 10.1093/gerona/glt093 PMC3779634 23902935

[cit0011] Cauley, J. A., Chalhoub, D., Kassem, A. M., & Fuleihan, G. E.-H. (2014). Geographic and ethnic disparities in osteoporotic fractures. *Nature Reviews. Endocrinology*, 10(6), 338–351. 10.1038/nrendo.2014.51 24751883

[cit0012] Clancy, A., Simonsen, N., Lind, J., Liveng, A., & Johannessen, A. (2020). The meaning of dignity for older adults: A meta-synthesis. *Nursing Ethics*, Jul 2:969733020928134. 10.1177/0969733020928134 32613895 PMC8408827

[cit0013] Coyne, I. T. (1997). Sampling in qualitative research. Purposeful and theoretical sampling; merging or clear boundaries? *Journal of Advanced Nursing*, 26(3), 623–630. 10.1046/j.1365-2648.1997.t01-25-00999.x 9378886

[cit0014] Cummings, S. R., & Melton, L. J. (2002). Epidemiology and outcomes of osteoporotic fractures. *The Lancet*, 359(9319), 1761–1767. 10.1016/S0140-6736(02)08657-9 12049882

[cit0015] Dennett, A. M., Taylor, N. F., & Mulrain, K. (2012). Community ambulation after hip fracture: Completing tasks to enable access to common community venues. *Disability and Rehabilitation*, 34(9), 707–714. 10.3109/09638288.2011.615371 22010644

[cit0016] Dyer, S. M., Crotty, M., Fairhall, N., Magaziner, J., Beaupre, L. A., Cameron, I. D., & Sherrington, C. (2016). A critical review of the long-term disability outcomes following hip fracture. *BMC Geriatrics*, 16(1), 158. 10.1186/s12877-016-0332-0 27590604 PMC5010762

[cit0017] Fleming, V., Gaidys, U., & Robb, Y. (2003). Hermeneutic research in nursing: Developing a Gadamerian‐based research method. *Nursing Inquiry*, 10(2), 113–120. 10.1046/j.1440-1800.2003.00163.x 12755860

[cit0018] Fog, J. (1998). *Med samtalen som udgangspunkt* [Conversation as a starting point]. Akademisk forlag.

[cit0019] Gadamer, H. G. (2013). *Truth and method* (2nd ed.). Bloomsbury Academy Publisher.

[cit0020] Gadamer, H.-G. (1996). *The enigma of health* (J. Gaiger & N. Walker, Trans.). Stanford University Press.

[cit0021] Galvin, K., & Todres, L. (2012). *Caring and well-being: A lifeworld approach*. Routledge.

[cit0022] Galvin, K. T., & Todres, L. (2011). Kinds of well-being: A conceptual framework that provides direction for caring. *International Journal of Qualitative Studies on Health and Well-being*, 6(4), 10362. 10.3402/qhw.v6i4.10362 PMC3235360 22171222

[cit0023] Gesar, B., Baath, C., Hedin, H., & Hommel, A. (2017). Hip fracture; an interruption that has consequences four months later. A qualitative study. *International Journal of Orthopaedic and Trauma Nursing*, Aug;26:43–48. 10.1016/j.ijotn.2017.04.002 28527900

[cit0024] Griffiths, F., Mason, V., Boardman, F., Dennick, K., Haywood, K., Achten, J., Parsons, N., Griffin, X., & Costa, M. (2015). Evaluating recovery following hip fracture: A qualitative interview study of what is important to patients. *BMJ Open*, Jan 6;5(1), e005406–2014. 10.1136/bmjopen-2014-005406 PMC4289715 25564138

[cit0025] Handoll, H. H. G., Sherrington, C., & Mak, J. (2011). *Interventions for improving mobility after hip fracture surgery in adults*. The Cochrane Library. 10.1002/14651858.CD001704.pub4 21412873

[cit0026] Healee, D. J., McCallin, A., & Jones, M. (2011). Older adult’s recovery from hip fracture: A literature review. *International Journal of Orthopaedic and Trauma Nursing*, 15(1), 18–28. 10.1016/j.ijotn.2010.06.010 28416177

[cit0027] Heidegger, M. (1962). *Being and time. 1927*. Harper Perrennial.

[cit0028] Heidegger, M. (1971). Building dwelling thinking. *Poetry, Language, Thought*, *154,* 1-26.

[cit0029] Holt-Lunstad, J., Smith, T. B., & Layton, J. B. (2010). Social relationships and mortality risk: A meta-analytic review. *PLoS Medicine*, 7(7), e1000316. 10.1371/journal.pmed.1000316 20668659 PMC2910600

[cit0030] Jellesmark, A., Herling, S. F., Egerod, I., & Beyer, N. (2012). Fear of falling and changed functional ability following hip fracture among community-dwelling elderly people: An explanatory sequential mixed method study. *Disability and Rehabilitation*, 34(25), 2124–2131. 10.3109/09638288.2012.673685 22536796

[cit0031] Kanis, J. A., Odén, A., McCloskey, E. V., Johansson, H., Wahl, D. A., & Cooper, C. (2012). A systematic review of hip fracture incidence and probability of fracture worldwide. *Osteoporosis International*, 23(9), 2239–2256. 10.1007/s00198-012-1964-3 22419370 PMC3421108

[cit0032] Lee, D. C., Sui, X., Ortega, F. B., Kim, Y. S., Church, T. S., Winett, R. A., Ekelund, U., Katzmarzyk, P. T., & Blair, S. N. (2011). Comparisons of leisure-time physical activity and cardiorespiratory fitness as predictors of all-cause mortality in men and women. *British Journal of Sports Medicine*, 45(6), 504–510. 10.1136/bjsm.2009.066209 20418526

[cit0033] Magaziner, J., Chiles, N., & Orwig, D. (2015). Recovery after hip fracture: Interventions and their timing to address deficits and desired outcomes-evidence from the Baltimore Hip Studies. *Nestle Nutrition Institute Workshop Series*, 83: 71–81.doi: 10.1159/000382064. 26484873 10.1159/000382064 PMC5494960

[cit0034] Norlyk, A., Haahr, A., & Hall, E. (2016). Interviewing with or without the partner present? –An underexposed dilemma between ethics and methodology in nursing research. *Journal of Advanced Nursing*, 72(4), 936–945. 10.1111/jan.12871 26708729

[cit0035] Pan, E., Bloomfield, K., & Boyd, M. (2019). Resilience, not frailty: A qualitative study of the perceptions of older adults towards “frailty”. *International Journal of Older People Nursing*, 14(4), e12261. 10.1111/opn.12261 31373440

[cit0036] Peeters, C. M. M., Visser, E., Van de Ree, C. L. P., Gosens, T., Den Oudsten, B. L., & De Vries, J. (2016). Quality of life after hip fracture in the elderly: A systematic literature review. *Injury*, 47(7), 1369–1382. 10.1016/j.injury.2016.04.018 27178770

[cit0037] Price, B. (2002). Laddered questions and qualitative data research interviews. *Journal of Advanced Nursing*, 37(3), 273–281. 10.1046/j.1365-2648.2002.02086.x 11851798

[cit0038] Puterbaugh, J. S. (2009). The emperor’s tailors: The failure of the medical weight loss paradigm and its causal role in the obesity of America. *Diabetes, Obesity & Metabolism*, 11(6), 557–570. 10.1111/j.1463-1326.2009.01019.x 19383033

[cit0039] Rasmussen, B., Nielsen, C. V., & Uhrenfeldt, L. (2018). Being active after hip fracture; older people’s lived experiences of facilitators and barriers. *International Journal of Qualitative Studies on Health and Well-being*, 13(1), 1554024. 10.1080/17482631.2018.1554024 30704373 PMC6327563

[cit0040] Rasmussen, B., Nielsen, C. V., & Uhrenfeldt, L. (2020). Being active 1½ years after hip fracture: A qualitative interview study of aged adults’ experiences of meaningfulness. *BMC Geriatrics*, 20(1), 263. 10.1186/s12877-020-01666-w 32727376 PMC7391487

[cit0041] Rasmussen, B., & Uhrenfeldt, L. (2016). Establishing well-being after hip fracture: A systematic review and meta-synthesis. *Disability and Rehabilitation*, 38(26), 2515–2529. 10.3109/09638288.2016.1138552 26860402

[cit0042] Roche, J. J., Wenn, R. T., Sahota, O., & Moran, C. G. (2005). Effect of comorbidities and postoperative complications on mortality after hip fracture in elderly people: Prospective observational cohort study. *BMJ*, 331(7529), 1374. 10.1136/bmj.38643.663843.55 16299013 PMC1309645

[cit0043] Shenton, A. K. (2004). Strategies for ensuring trustworthiness in qualitative research projects. *Education for Information*, 22(2), 63–75. 10.3233/EFI-2004-22201

[cit0044] Sherrington, C., Whitney, J. C., Lord, S. R., Herbert, R. D., Cumming, R. G., & Close, J. C. T. (2008). Effective exercise for the prevention of falls: A systematic review and meta‐analysis. *Journal of the American Geriatrics Society*, 56(12), 2234–2243. 10.1111/j.1532-5415.2008.02014.x 19093923

[cit0045] Sims-Gould, J., Stott-Eveneshen, S., Fleig, L., McAllister, M., & Ashe, M. C. (2017). Patient perspectives on engagement in recovery after hip fracture: A qualitative study. *Journal of Aging Research*, 2017;2017:2171865. 10.1155/2017/2171865 PMC5376933 28409031

[cit0046] Smythe, E. A., Ironside, P. M., Sims, S. L., Swenson, M. M., & Spence, D. G. (2008). Doing Heideggerian hermeneutic research: A discussion paper. *International Journal of Nursing Studies*, 45(9), 1389–1397. 10.1016/j.ijnurstu.2007.09.005 17950738

[cit0047] Tak, E., Kuiper, R., Chorus, A., & Hopman-Rock, M. (2013). Prevention of onset and progression of basic ADL disability by physical activity in community dwelling older adults: A meta-analysis. *Ageing Research Reviews*, 12(1), 329–338. 10.1016/j.arr.2012.10.001 23063488

[cit0048] Tang, V. L., Sudore, R., Cenzer, I. S., Boscardin, W. J., Smith, A., Ritchie, C., Wallhagen, M., Finlayson, E., Petrillo, L., & Covinsky, K. (2017). Rates of recovery to pre-fracture function in older persons with hip fracture: An observational study. *Journal of General Internal Medicine*, 32(2), 153–158. 10.1007/s11606-016-3848-2 27605004 PMC5264672

[cit0049] Taylor, N. F., Barelli, C., & Harding, K. E. (2010). Community ambulation before and after hip fracture: A qualitative analysis. *Disability and Rehabilitation*, 32(15), 1281–1290. 10.3109/09638280903483869 20156052

[cit0050] Todres, L., & Galvin, K. (2010). “Dwelling-mobility”: An existential theory of well-being. *International Journal of Qualitative Studies on Health and Well-being*, 5(3), 5444. 10.3402/qhw.v5i3.5444 PMC2938924 20842215

[cit0051] Tong, A., Sainsbury, P., & Craig, J. (2007). Consolidated criteria for reporting qualitative research (COREQ): A 32-item checklist for interviews and focus groups. *International Journal for Quality in Health Care*, 19(6), 349–357. 10.1093/intqhc/mzm042 17872937

[cit0052] Visschedijk, J., Van Balen, R., Hertogh, C., & Achterberg, W. (2013). Fear of falling in patients with hip fractures: Prevalence and related psychological factors. *Journal of the American Medical Directors Association*, 14(3), 218–220. 10.1016/j.jamda.2012.10.013 23218746

[cit0053] Vogel, T., Brechat, P. H., Leprêtre, P. M., Kaltenbach, G., Berthel, M., & Lonsdorfer, J. (2009). Health benefits of physical activity in older patients: A review. *International Journal of Clinical Practice*, 63(2), 303–320. 10.1111/j.1742-1241.2008.01957.x 19196369

[cit0054] Whipple, M. O., Hamel, A. V., & Talley, K. M. C. (2018). Fear of falling among community-dwelling older adults: A scoping review to identify effective evidence-based interventions. *Geriatric Nursing*, 39(2), 170–177. 10.1016/j.gerinurse.2017.08.005 28941942 PMC5862787

[cit0055] Wiles, J. L., Wild, K., Kerse, N., & Allen, R. E. S. (2012). Resilience from the point of view of older people:‘There’s still life beyond a funny knee’. *Social Science & Medicine*, 74(3), 416–424. 10.1016/j.socscimed.2011.11.005 22204841

[cit0056] World Medical Association Declaration of Helsinki. (2013). World Medical Association Declaration of Helsinki: Ethical principles for medical research involving human subjects. *Jama*, 310(20), 2191–2194. 10.1001/jama.2013.281053 24141714

[cit0057] Young, Y., & Resnick, B. (2009). Don’t worry, be positive: Improving functional recovery 1 year after hip fracture. *Rehabilitation Nursing*, 34(3), 110–117. 10.1002/j.2048-7940.2009.tb00265.x 19475806 PMC2729270

[cit0058] Ziden, L., Scherman, M. H., & Wenestam, C. G. (2010). The break remains-Elderly people’s experiences of a hip fracture 1 year after discharge. *Disability and Rehabilitation: An International, Multidisciplinary Journal*, 32(2), 103–113. 10.3109/09638280903009263 19562584

[cit0059] Ziden, L., Wenestam, C. G., & Hansson-Scherman, M. (2008). A life-breaking event: Early experiences of the consequences of a hip fracture for elderly people. *Clinical Rehabilitation*, 22(9), 801–811. 10.1177/0269215508090204 18728133

[cit0060] Zusman, E. Z., Dawes, M. G., Edwards, N., & Ashe, M. C. (2018). A systematic review of evidence for older adults’ sedentary behavior and physical activity after hip fracture. *Clinical Rehabilitation*, 32(5), 679–691. 10.1177/0269215517741665 29169245

